# Human placental extract suppresses mast cell activation and induces mast cell apoptosis

**DOI:** 10.1186/s13223-023-00850-y

**Published:** 2023-11-27

**Authors:** Tongqian Wu, Jingjing He, Shirong Yan, Jing Li, Ke Chen, Dingshan Zhang, Mingliang Cheng, Zou Xiang, Yu Fang

**Affiliations:** 1 Center for Clinical Laboratories, Affiliated Hospital of Guizhou Medical University, Guiyi Street 28, Guiyang, 550004 Guizhou China; 2 School of Laboratory Science, Guizhou Medical University, Guiyang, China; 3 Clinical Research Center, Affiliated Hospital of Guizhou Medical University, Guiyang, China; 4 Department of Infectious Disease, Affiliated Hospital of Guizhou Medical University, Guiyi Street 28, Guiyang, 550004 Guizhou China; 5 Department of Health Technology and Informatics, Faculty of Health and Social Science, The Hong Kong Polytechnic University, Hong Kong, China

**Keywords:** Mast cell, Allergy, Human placental extract, Passive cutaneous anaphylaxis, Mast cell activation, Degranulation

## Abstract

**Background:**

Human placental extract (HPE) has been documented to facilitate the healing of certain disorders including allergy. However, the effects of HPE on the functionality of mast cells, a critical cell type in allergic diseases, have not been reported.

**Methods:**

To investigate the effects of HPE on the regulation of allergy with respect to the biological functions of mast cells, the mast cell line C57 or HMC-1 cells were treated with HPE followed by the assessment of cell proliferation, apoptosis, activation, chemotaxis and phagocytosis. Mouse peritoneal mast cells were also investigated for their responses to induction of apoptosis by HPE in vivo. Furthermore, the effect of HPE on mast cell degranulation was confirmed using the passive cutaneous anaphylaxis (PCA) assay, an acute allergy model.

**Results:**

HPE was capable of suppressing mast cell proliferation and inducing mast cell apoptosis. Mast cell degranulation in response to compound 48/80- or anti-DNP IgE and DNP-mediated activation was suppressed. In addition, treatment with HPE compromised the production of cytokines by mast cells and cell chemotaxis. These observations were consistent with the dampened passive cutaneous anaphylaxis (PCA) assay following treatment with HPE.

**Conclusion:**

This study revealed a suppressive effect of HPE on overall mast cell activities, suggesting a potential regulatory role of HPE on the alleviation of allergic diseases through mast cells.

**Supplementary Information:**

The online version contains supplementary material available at 10.1186/s13223-023-00850-y.

## Background

The prevalence of allergic diseases, including asthma, rhinitis, eczema and food allergies, is now much higher than during the past half-century. More than 30% of children worldwide are suffering from a form of allergy [1]. Currently more than 300 million people experience asthma attacks globally, and this number may likely be increased by another one third by 2025 [2]. The characteristics and consequences of acute and chronic allergic inflammation have been extensively documented, including the key contributors involved in the development of the diseases. Mast cells have long been considered critical effector cells in allergy because of their potential to secrete a variety of allergic mediators [3]. Mast cell density in tissues correlates with the severity of allergic symptoms [4]. Strategies targeting mast cells to prevent or alleviate the provocation of allergic reactions have been extensively investigated based on findings that reveal mechanisms by which mast cells contribute to allergy [5]. Mast cells are non-circulating immune cells that develop only when bone marrow-derived precursors have reached their target tissue [6].

Mast cells are important effector and regulatory cells in allergic diseases. Activation and recruitment of mast cells are important effector processes in the onset and development of allergic diseases [7]. Mast cells are innate immune cells that serve as sentinels within tissues exposed to external environment and release a multitude of mediators that coordinate the immune responses. However, the aberrant accumulation and activation of mast cells can result in progression of inflammatory disorders [8].

Human placental extract (HPE) are small-molecule active peptides extracted from the placenta, which contain a variety of biologically active substances such as immunoglobulins, bioactive peptides and hormones, as well as amino acids and minerals. HPE has been used as a traditional therapeutic agent in many countries for the supplemental treatment of certain diseases and the replenishment of significance [9–11]. Specifically, HPE has been used widely for dampening inflammation, improvement of fatigue, anti-aging, wound healing and pain relief [12, 13]. Doctors chose direct application of HPE as their priority for burned patients to both prevent infection and promote healing [14]. The placental extract is useful for the maintenance of human skin quality [15], improvement of sleep quality [16], reduction of menopausal symptoms [17], and maintenance of joint functions in knee osteoarthritis patients [18]. Multiple studies have demonstrated that HPE is also involved in immune regulation. HPE retards graft-versus-host diseases (GVHD) which are associated with T lymphocytes by inducing the redistribution of regulatory T (Treg) cells [19, 20]. HPE suppresses allergic airway inflammation and potentiate induction of regulatory T cells in a murine model of allergic rhinitis [20]. However, despite its popularity in use as a therapeutic agent, the efficacy and mechanisms of action of HPE have not been sufficiently studied. Given that mast cells are a major contributor in allergic and inflammatory disorders, it was our interest to investigate the effects of HPE on mast cell biology. Therefore, in this study, we undertook to explore the regulatory effects of HPE on mast cell functionality by using a mast cell line and validation in relevant mouse models.

## Materials and methods

### Animals

Mice of the C57BL/6 and Balb/c strains were purchased from Charles River (Beijing, China) and were bred in-house at the experimental animal facility of the Affiliated Hospital of Guizhou Medical University. Male mice which were 8–10 weeks old were used at the start of all experiments. All the mice were housed together under specific pathogen-free conditions at the experimental animal facility for at least 2 weeks before the experiments. The animal protocols were approved by the Ethics Committee of the Guizhou Medical University.

### Human placental extract

The human placental extract (HPE) used in this study was a commercial medicine (H20046260, Taibang Health, Guizhou, China) extracted from post-partum/post-natal placental tissues, and it has been routinely applied as supplemental therapy on demand in the clinic. The State Food and Drug Administration (SFDA) of China approval number of HPE is H20046260. The lyophilized powder of HPE was dissolved in sterile phosphate buffer saline (PBS) in the experiments.

### Cell line and culture

The mouse-derived mast cell line (C57) was obtained from Dr. Gunnar Nilsson, Karolinska Institute, Sweden). The human-derived mast cell line (HMC-1) was provided by Stem Cell Bank, Chinese Academy of Sciences. C57 cells were cultured in RPMI 1640 medium supplemented with 10% heat-inactivated fetal bovine serum (Gibco, Grand Island, USA), 4 mmol/L L-glutamine (Gibco), 50 μmol/L 2-mercaptoethanol (Sigma-Aldrich, USA) and 100 μg/mL penicillin/streptomycin (Gibco). HMC-1 cells were cultured in Iscove’s Modified Dulbecco’s Medium supplemented with 10% heat-inactivated fetal bovine serum and 2 mmol/L L-glutamine.

### Cell proliferation and viability assay

The effect of HPE on cell viability was assessed using a Cell Counting Kit-8 (CCK-8, Dojindo Molecular Technologies, Osaka, Japan) assay. Briefly, C57 or HMC-1 cells (1 × 10^4^ cells/well) were seeded in a 96-well plate and treated with various concentrations of HPE or PBS and were incubated at 37 °C for 4, 8 or 16 h. The CCK-8 reagent was added for the last 4 h for assessing the value of half maximal inhibitory concentration (IC50) or the proliferative capacity of cells. Or alternatively cells were fixed and permeabilized (BD Cytofix/Cytoperm, BD Biosciences) followed by stained with FITC-conjugated Ki-67 (Clone; 11F6, Biolegend, San Diego, California, USA) and analyzed with a flow cytometer (Navios, Beckman coulter, Brea, California, USA).

### Apoptosis assay

Cell apoptosis was measured by flow cytometry to determine the profile of annexin V (BD Biosciences, Franklin Lake, New Jersey, USA) and propidium iodide (PI, BD Biosciences). In brief, 2.5 × 10^5^ C57 or HMC-1 cells were incubated with various concentrations of HPE for 4, 8 or 16 h. Next, cells were stained with FITC-annexin V/PI for 20 min followed by analysis by a flow cytometer (Navios).

For measurement of mast cell apoptosis in vivo, C57BL/6 mice were challenged i.p with 120 ng HPE or an identical volume of PBS (5 mice per group) i.p. for 4, 8 or 16 h. Next, 5 mL PBS was injected into the euthanized mouse peritoneal cavity and peritoneal lavage fluid (PLF) was harvested after 5 min. The PLF cells were stained with BV421-conjugated rat anti-mouse CD117 (cKit) (Clone; 2B8, Biolegend, San Diego, CA), APC- conjugated rat anti-mouse FcεRIα (Clone; MAR-1, Biolegend) and FITC-conjugated annexin V/PI (BD Biosciences) and examined by a flow cytometer (Navios).

### Degranulation assay

C57 or HMC-1 cells (2.5 × 10^5^) were pre-treated with 30 ng/mL of HPE for 4, 8 h, and HPE was next removed by extensive washing. Cells were activated with 1.5 μg/mL compound 48/80 (C48/80, Sigma-Aldrich, St. Louis, MO) for 30 min in serum-free medium. Alternatively, cells were treated with 1 μg/mL anti-DNP IgE (clone SPE-7; Sigma-Aldrich) for 4, 8 h and were subsequently washed and challenged with 100 μg/mL DNP_-30–40_-HSA (Sigma-Aldrich) for 30 min at 37 °C in serum-free medium. The supernatant was collected for β-hexosaminidase (β-hex) measurement as a readout for mast cell degranulation. Briefly, the supernatant was mixed with an identical volume of 4-nitrophenyl N-acetyl-b-D-glucosaminide (Sigma Aldrich), the substrate of β-hex, and incubated for 1 h at 37 °C. The reaction was stopped by the addition of an equal volume of 0.2 M glycine (Sigma-Aldrich) (pH10). The absorbance at 405 nm was measured using a microplate reader [21].

### Cytokine measurement

C57 or HMC-1 cells were pre-treated with 30 ng/mL HPE or PBS for 4, 8 or 16 h and were subsequently washed. The cells were then treated with 1.5 μg/mL C48/80 for 3 h and the supernatant was collected. The concentrations of IL-4, IL-6, IL-13 and TNF-α in the supernatant were determined using a cytometric bead array assay (LEGENDplex^™^ Mouse Inflammation Panel, Biolegend) according to the manufacturer’s instruction. The fluorescence intensity was assessed on a Navios flow cytometer (Beckman, USA), followed by data analysis using LEGENDplex v8.0 software (Biolegend).

### Antigen phagocytosis

C57 or HMC-1 cells (2.5 × 10^5^ cells/well) were seeded in a 96-well plate and pre-treated with various concentrations of HPE for 0, 4, 8 or 16 h. After removal of HPE, cells were incubated with Alexa Fluor 488-conjugated OVA (Sigma-Aldrich) for 2 h followed by staining with APC-conjugated anti-mouse FcεRIα (Clone; MAR-1, Biolegend) and then analyzed by flow cytometry or a confocal laser scanning microscope (Zeiss, Jena, Germany), and the fluorescence intensity of engulfed OVA was analyzed by Image J software (National Institute of Mental Health, Bethesda, Maryland, USA).

### Chemotaxis assay

C57 or HMC-1 cells were pre-treated with 30 ng/mL HPE or PBS for 4, 8 or 16 h and were subsequently washed, and then were added to 8 μm fibronectin-coated transwells (VWR International, Radnor, PA) at 2 × 10^6^ cells per mL in 200 μL 10% medium with serum. Medium (600 μL) was added to the lower chambers with 25 ng/mL stem cell factor (SCF) [8]. Migration was carried out for 16 h at 37 °C. Plates were then shaken for 10 min to remove adherent cells from the bottom of the transwells [22]. Transwells were removed and DAPI (Solarbio, Beijing, China) was added to each well and cells were incubated at room temperature for 10 min. Cells were observed by a fluorescence microscope (Zeiss) and calculated using Image J software (National Institute of Mental Health).

### Passive cutaneous anaphylaxis assay

Anesthetized BALB/c mice were injected i.d. with 100 ng HPE in the right ear and with an identical volume of PBS in the left ear as a control for the traumatic response representing the result of skin injury. After 4, 8 or 16 h, 0.4 μg IgE directed against DNP in 10 μL PBS was i.d injected into both ears of a lightly anesthetized mouse. The PCA reaction was induced 24 h later by an intravenous injection of 10 μg DNP-_30–40_-HSA and 2% Evans blue in 200 μL of PBS. The euthanized mouse ears were removed 30 min later and the dye extravasation was quantified as previously described [21] with slight modification. In brief, ears were ground by a tissue homogenizer with 1 mL PBS. The exudate was collected and mixed with acetone (3:7, v/v), and incubated at room temperature overnight. After vigorous vortexing, the mixture was centrifuged at 3000 rpm for 15 min. The supernatant was collected for the measurement of extravasated Evans blue with an ELISA reader at 620 nm.

For some of the PCA assays, Evans blue was obviated and ear tissues were sectioned and analyzed with toluidine blue staining for revealing mast cell morphology as previously described [23]. Or alternatively, the ear sections were processed for histamine analysis as previously described [24]. Briefly, the sections were incubated with the rabbit anti-histamine antibody (Fine Test, Wuhan, China), and then with horseradish peroxidase-linked anti-rabbit and anti-mouse universal secondary antibodies (Solarbio, Beijing, China). The sections were counterstained with hematoxylin staining solution (Solarbio, Beijing, China) and photographed under a positive position microscope (Leica, Weztlar, Germany). The relative histamine expression was analysis with Image J software (National Institute of Mental Health).

### Statistical analyses

Statistical analyses were performed using GraphPad Prism software version 9.5.0 (GraphPad, San Diego, California, USA). Statistical analysis was performed using unpaired Student *t*-test, one-way ANOVA with Tukey’s multiple comparison test or two-way ANOVA with Tukey’s multiple comparison test. A *P* value < 0.05 is considered statistically significant.

## Results

### HPE inhibits mast cell proliferation and induces mast cell apoptosis

**Fig. 1:**
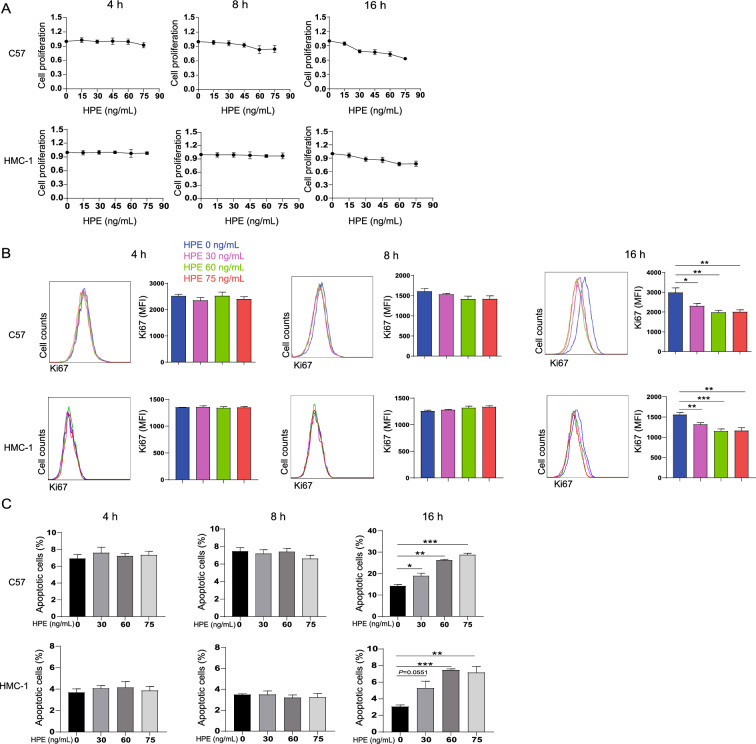
Human placental extract (HPE) inhibits proliferation and induces apoptosis of mast cells. **A** C57 or HMC-1 cells were treated with human placental extract (HPE) for 4, 8 or 16 h followed by incubation with CCK-8. Color development was analyzed by microplate reader and cell proliferation is shown as O.D values. **B**, **C** 2.5 × 10^5^ of C57 or HMC-1 cells were incubated in the absence or presence of HPE at various concentrations as indicated for 4, 8 or16 h, following with Ki-67 detection by flow cytometric analysis. The flow cytometric histograms and mean fluorescence intensity (MFI) was shown in **B**. Or cell apoptosis was analyzed by flow cytometric analysis in **C**. Data are shown as mean ± SEM (n = 3) of three separate experiments. *P < 0.05, **P < 0.01, ***P < 0.001, using the one-way ANOVA with Tukey’s multiple comparison test for statistical significance

Mast cells, especially connective tissue mast cells, have a long life span [25], and mature mast cells are still capable of proliferation [26, 27]. Therefore, regulation of mast cell proliferation may impact mast cell tissue density. The half maximal inhibitory concentration (IC50) of HPE was 79.94 ng/mL revealed by the CCK-8 analysis, so we further investigated whether HPE has an impact on the proliferation of mast cells within the IC50 range. After treatment with HPE at different concentrations for 4 or 8 h, the proliferation of C57 or HMC-1 mast cells remained unchanged; extending the HPE incubation to 16 h resulted in dose-dependent suppression of proliferation (Fig. 1A, B).

**Fig. 2:**
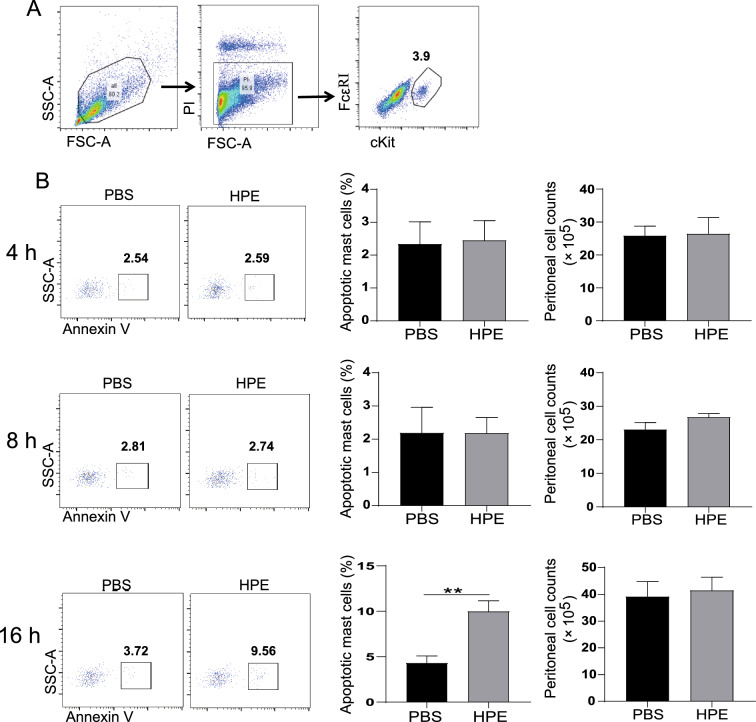
Human placental extract (HPE) induces apoptosis of peritoneal mast cells. Mice (BALB/c) were challenged intraperitoneally with 120 ng HPE or identical volume of PBS. Peritoneal lavage fluid was harvested after 4, 8 or 16 h and flow cytometry analysis the apoptosis of mast cell. **A** An example is given to show the gating strategy for cKit^+^/FcεRI^+^ peritoneal mast cells. The numbers adjacent to the boxed areas indicate percentage of cells in each. Mast cells apoptosis was defined as annexin V^+^ (left panel), and the percentages of apoptotic cells were plotted. **B** The apoptotic peritoneal mast cells and total numbers of peritoneal cells (right panel) from mice pre-treated with HPE for 4, 8 or 16 h. Data are shown as mean ± SEM (n = 5) of three separate experiments. **P < 0.01, using the unpaired Student *t* test for statistical significance

Apoptosis may critically shape tissue persistence of mast cells. Therefore, we next analyzed the induction of apoptosis of mast cells after treatment with various concentrations of HPE. Frequencies of apoptotic mast cells were dose-dependently elevated in response to HPE treatment for 16 h (Fig. 1C, with gating strategies shown in Additional file 1: Fig. S1), whereas no obvious apoptosis was observed at 4 or 8 h. Furthermore, HPE-mediated mast cell apoptosis was confirmed in vivo. Mice were thus injected with HPE in the peritoneal cavity and PLF was harvested. Peritoneal mast cells were identified based on the expression of cKit and FcεRI (Fig. 2A). Increased percentages of apoptotic peritoneal mast cells were observed following in vivo treatment with HPE for 16 h (Fig. 2B), which was consistent with the in vitro observation (Fig. 1C). No obvious changes of the frequencies of total PI^+^ cells were observed in response to HPE-treatment and approximately 3–5% of the PI^+^ cells are mast cells (Additional file 2: Fig. S2). HPE did not modulate the total numbers of peritoneal mast cells during the short incubation period (Fig. 2B), which supported the validity of the percentage comparison between naïve mice and mice treated with HPE.

### HPE suppresses mast cell degranulation

**Fig. 3:**
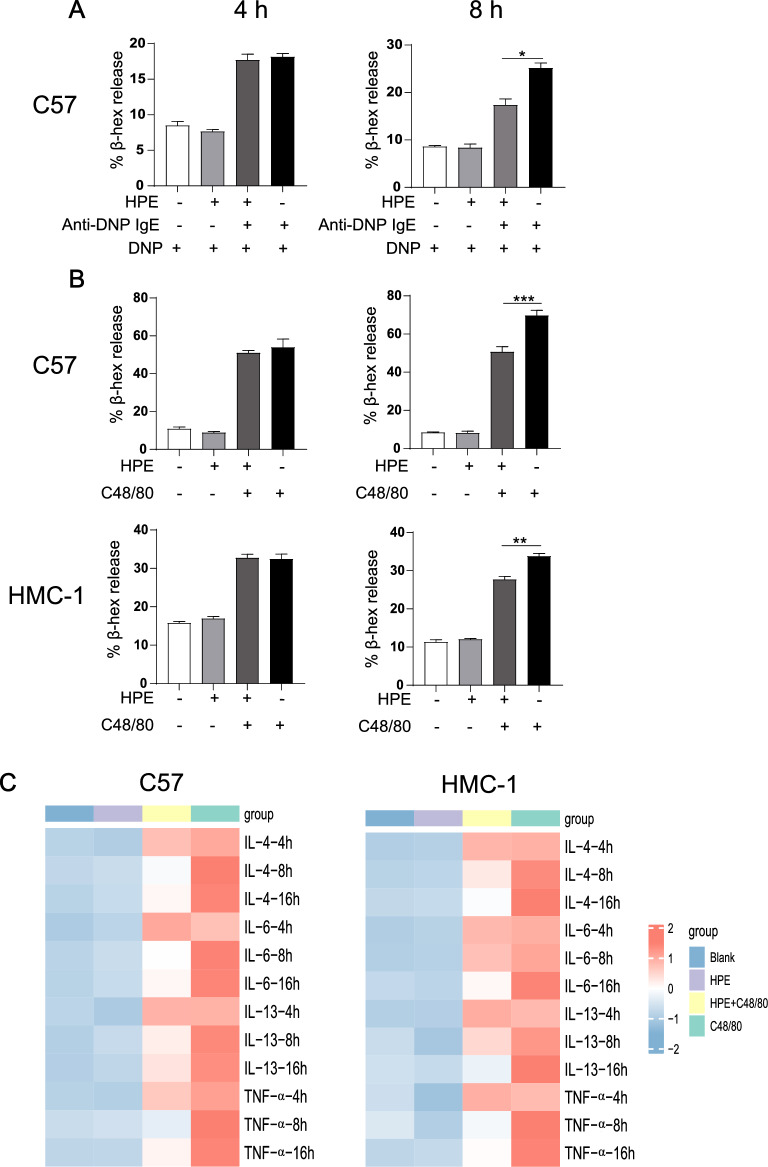
Human placental extract (HPE) suppresses activation of mast cell following treatment with C48/80 or IgE/anti-IgE. C57 or HMC-1 cells were pre-treated with or without HPE (30 ng/mL) for 4, 8 or 16 h followed by washing. Cells were next subjected to treatment with anti-DNP IgE or activation through C48/80 as indicated. **A** β-hexosaminidase (β-hex) was measured in culture supernatant with anti-DNP IgE after pre-treatment by HPE for 4, 8 h. **B** β-hexosaminidase (β-hex) was measured in culture supernatant with C48/80 after pre-treatment by HPE for 4, 8 h. **C** Cytokines of IL-4, IL-6, IL-13 or TNF-α were analyzed in culture supernatant using cytometric bead array analysis after treatment with C48/80 for 4, 8 or 16 h. Heatmap of cytokine release was shown. Data are shown as mean ± SEM (n = 3) of three separate experiments.*P < 0.05, **P < 0.01, ***P < 0.001, using the two-way ANOVA with Tukey’s multiple comparison test for statistical significance

Mast cells regulate allergic and inflammatory responses through secreting a variety of allergic mediators upon activation. Therefore, we next investigated the effect of HPE on mast cell activation. Since HMC-1 cells lack high affinity IgE receptor FcεRI [28], so the effect of HPE on the IgE-induced classic activation was only performed on C57 cells. HPE alone failed to stimulate mast cell degranulation measured as β-hex release (Fig. 3A, B). Mast cell activation was readily initiated following either treatment with C48/80, a mast cell activator, or by IgE receptor crosslinking, the classical mast cell activation pathway (Fig. 3A, B). Interestingly, pre-treatment with HPE substantially suppressed degranulation of mast cells triggered by C48/80 or IgE receptor crosslinking (Fig. 3A, B), suggesting an inhibitory effect of HPE on mast cell degranulation. Specifically, the suppressive effect of HPE on the mast cell activation was observed when the cells were pre-treatment with HPE for 8 h, the timepoint when no obvious increase of apoptotic cells was presented both in vitro (Fig. 1C) and in vivo (Fig. 2B). In addition to degranulation, mast cell activation is also accompanied by cytokine production. We, therefore, next evaluated the modulation of cytokine production profile of mast cells by HPE. Decreased levels of cytokines, including IL-4, IL-6, IL-13 and TNF-α, were observed in both C57 or HMC-1 mast cells in response to C48/80 after pre-incubation with HPE (Fig. 3C), among which dampened levels of IL-4 and TNF-α were already observed at the 8 h-timepoint (Fig. 3C), supporting an inhibitory effect of HPE on cytokine production by mast cells.

### Antigen phagocytosis by mast cells is not modulated by HPE

**Fig. 4:**
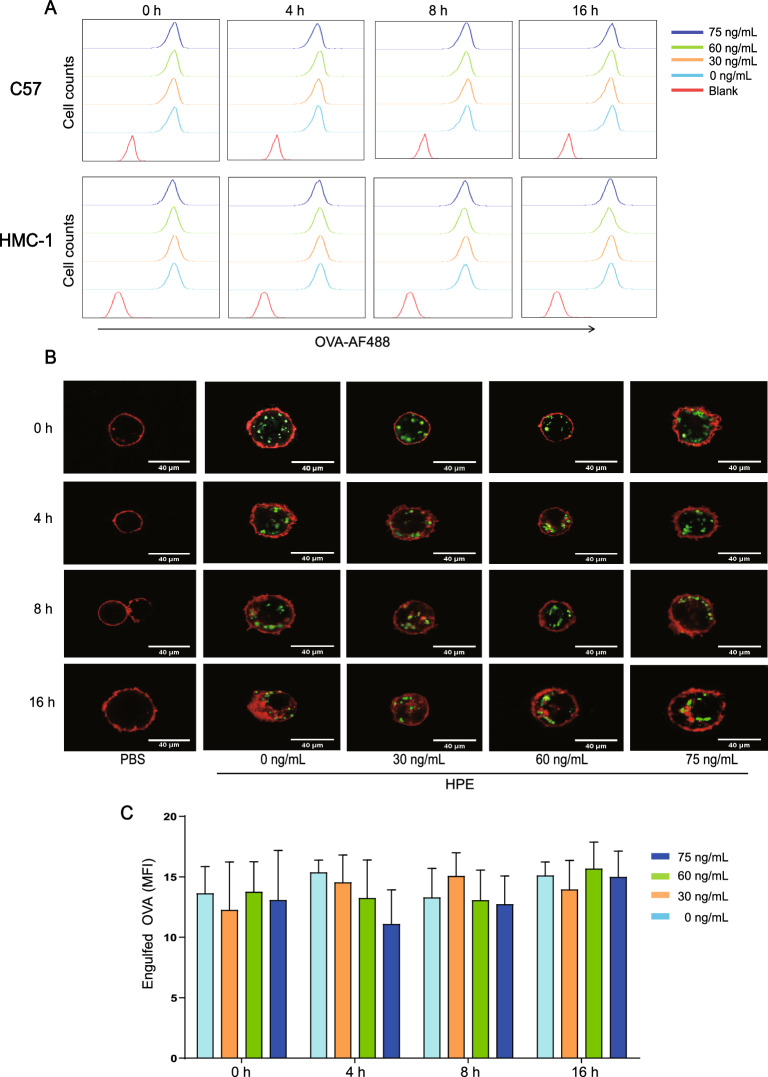
Antigen phagocytosis by mast cells is not affected by human placental extracts (HPE). C57 or HMC-1 cells were treated with or without HPE at concentrations and durations as indicated followed by stimulation with 80 mg/mL of OVA-AF488 for 2 h. OVA uptake was assessed by flow cytometry (**A**) or a confocal microscope (**B**). The fluorescence intensity of engulfed OVA was pooled in (**C**). Data in (**C**) are presented as mean ± SEM (n = 3) of three separate experiments. Using the one-way ANOVA with Tukey’s multiple comparison test for statistical significance

Despite not being professional phagocytes, mast cells are capable of phagocytosis [29]. Therefore, we also assessed the effect of HPE on mast cell phagocytosis. We failed to find any evidence indicating a regulatory effect of HPE on phagocytosis of mast cells examined by flow cytometry (Fig. 4A) and con-focal microscopy (Fig. 4B, C).

### HPE inhibits mast cell chemotaxis towards SCF

**Fig. 5:**
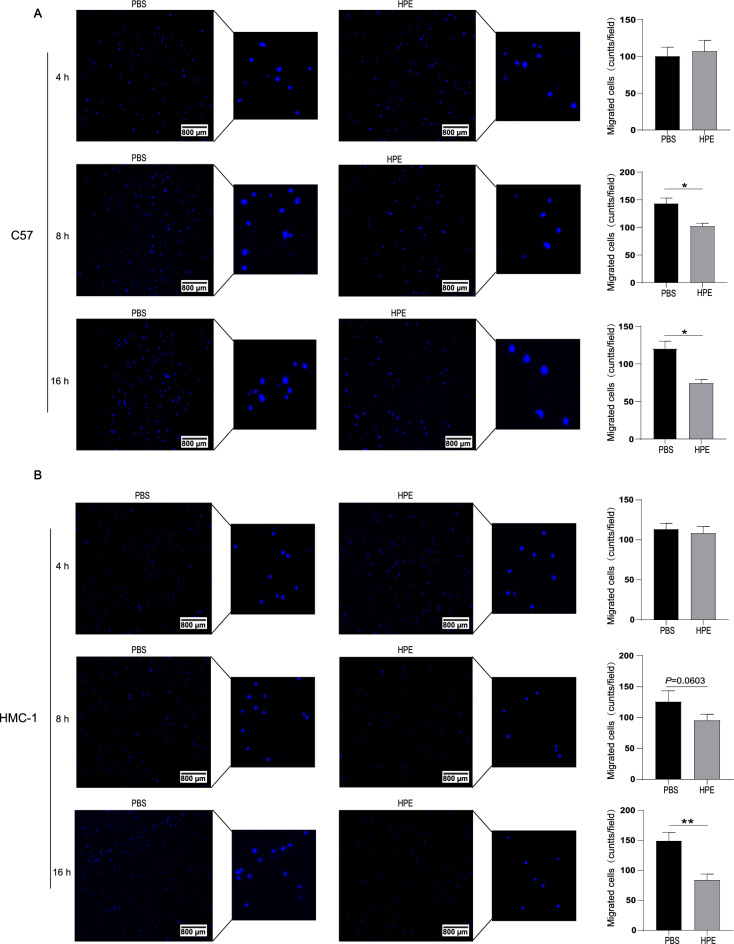
Human placental extract (HPE) attenuates the chemotaxis of C57 cells. A transwell assay was performed to measure C57 or HMC-1 cells chemotaxis toward SCF (25 ng/mL) after pre-treated with various concentration of HPE as indicated. **A** Cell migration was examined by a fluorescence microscopy. **B** Cells from multiple fields (n = 5) were tracked individually to calculate migrated cell numbers. Data are shown as mean ± SEM (n = 3) of three separate experiments. ** *P* < 0.01, **** *P* < 0.0001, using the unpaired Student *t* test for statistical significance

Mast cells not only rely on SCF for differentiation and maturation [30], but also migrate to SCF which serves as a chemotactic factor [31]. We evaluated the possible regulatory effect of HPE on SCF-mediated mast cell chemotaxis. Reduced numbers of C57 mast cells were chemo-attracted from the upper chamber towards the lower chamber containing SCF when cells had been pre-incubated with HPE for 8 or 16 h (Fig. 5A). HPE suppressed the migration of HMC-1 cells at 16 h and with an inhibitory trend at 8 h (Fig. 5B), together demonstrating an inhibitory effect of HPE on mast cell chemotaxis.

### HPE attenuates PCA responses of mice

**Fig. 6:**
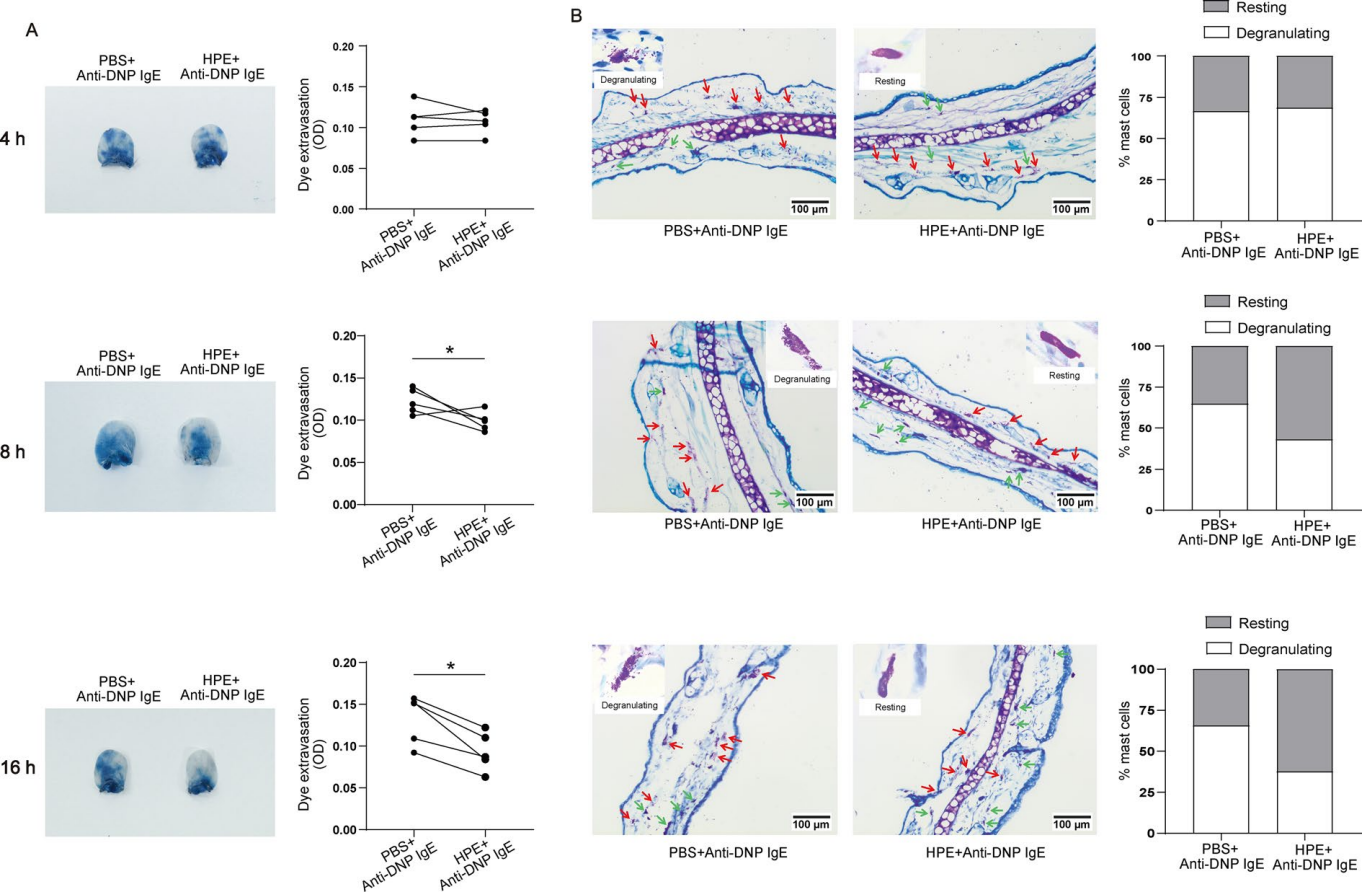
Human placental extract (HPE) suppresses passive cutaneous anaphylaxis reaction. The right ear of BALB/c mouse was injected i.d. with 100 ng HPE, and the left ear was injected with an identical volume of PBS. After 24 h, both ears received 0.4 μg IgE in 10 μL PBS. The PCA reaction was induced 24 h later by an intravenous injection of 10 μg DNP-30–40-HSA and 2% Evans blue in 200 μL PBS. Thirty minutes after the provocation, the ears were collected and processed by tissue homogenizer after the mouse were sacrificed. **A** 30 min after the dye injection, ears were excised and photographed (left panel, depicting ears from one mouse), and dye extravasation was quantified and plotted (right panels). Each line represents data from an individual mouse, and bars indicate the average values. **B** Alternatively, ear tissue sectioning was prepared. Toluidine blue staining was used to identify tissue mast cells. Resting or degranulating mast cells could be visualized. Green arrows, degranulating cells; red arrows, resting cells. Numbers of resting and degranulating mast cells in ear tissues were enumerated and plotted. The percentages of degranulating mast cells in the PBS-treated ears versus HPE–treated ears were 66.6 ± 12.18% versus 67.8 ± 14.7% (4 h-pretreatment), 64.8 ± 14.4% versus 34.1 ± 12.8% (8 h-pretreatment) and 65.7 ± 9.45% versus 37.7 ± 16.3% (16 h-pretreatment), respectively (five mice; mean ± SEM; *P* = 0.0004). Data are shown as mean ± SEM (n = 5) of three separate experiments.*P < 0.05, using the unpaired Student *t* test for statistical significance

The mouse PCA model is a measure of mast cell activation and degranulation in vivo [32]. Therefore, we also evaluated the effect of HPE on PCA in mice. Local IgE sensitization and systemic administration of DNP/Evans blue triggered mast cell activation evidenced by augmented dye extravasation in the ear tissue compared with the ear that had not received IgE (Fig. 6A). Dye extravasation, representing tissue mast cell degranulation, was suppressed in the ear that had received HPE for 8 h or 16 h, in addition to IgE (Fig. 6A, middle and below panels), and no difference was observed in the 4 h-pretreated ear (Fig. 6A, upper panel). Tissue sectioning of the ears was performed, and slides were stained with toluidine blue to specifically stain for mast cell granules, allowing discrimination of degranulating and resting mast cells as we previously reported [33]. We observed fewer degranulating mast cells in ears that being pre-treated with HPE for 8 h or 16 h, compared with those ears without HPE administration (Fig. 6B), which was consistent with the dye extravasation assay.

**Fig. 7:**
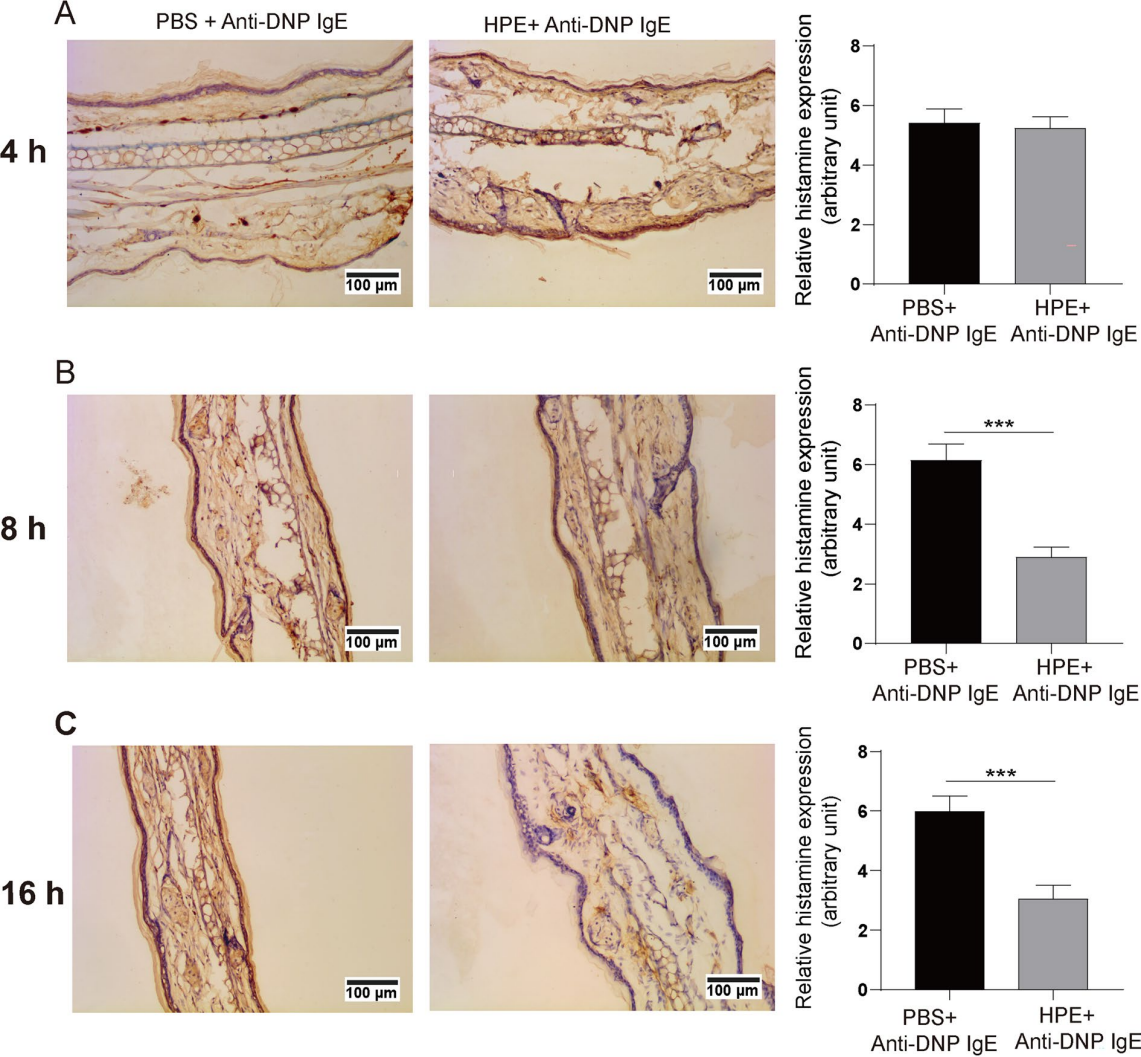
Human placental extract (HPE) suppresses histamine release in cutaneous anaphylaxis reaction. The PCA was induced in BALB/c mouse pre-treatment with HPE for 4 (**A**), 8 (**B**) or 16 h (**C**) as described in Fig. 6, and followed by peroxidase-based immunostaining using an anti-histamine antibody. The relative histamine expression was analysis with Image J software. The typical views were shown (left panels) and the relative histamine expression was quantified and plotted (right panels). At least five randomly chosen areas were scanned for determining the intensity of histamine which was expressed as optical density (OD) per area. Data are shown as mean ± SEM (n = 5) of three separate experiments. *** *P* < 0.001, using the unpaired Student *t* test for statistical significance

Histamine is the main effector produced by activated mast cells, and the variation of histamine in the local tissue could indirectly indicate the activation of mast cells [34]. Comparatively less histamine production, another indication of mast cell activation, was observed in the ear tissue that had received pre-treatment of HPE for 8 h or 16 h (Fig. 7A–C), together substantiating the inhibitory effect of HPE on tissue mast cell activation.

## Discussion

HPE has been traditionally exploited as a treatment regimen for inflammatory diseases [35]. Various HPE ingredients, including peptides, amino acids, and hormones [36], have been shown to be effective in different studies. HPE has been reported to exert a suppressive role in a murine allergic rhinitis model partly through induction of regulatory T cells [20]. We speculated that mast cell activities could also be modulated by HPE in allergy, as mast cells are important effector cells in allergy and remain one of the most crucial therapeutic targets for the alleviation of allergic diseases [3]. However, limited information is available regarding the impact of HPE on the biologic functions of mast cells. In the present study, we show that HPE suppressed mast cell activities, including proliferation, activation and chemotaxis as well as induced cell apoptosis.

We first demonstrated HPE-mediated retardation of mast cell proliferation. Placenta-derived molecules may exert different effects depending on the type of cells. It has been reported that placental indoleamine 2,3-dioxygenase-mediated tryptophan degradation can induce lymphocyte proliferation [37]. The seemingly opposite effect of HPE on mast cell proliferation observed in the present study may be dependent on other mechanisms which await further clarification.

In addition to proliferation, apoptosis can also affect tissue persistence of mast cells, which has a documented impact on the development of allergic diseases. We demonstrated that HPE induced apoptosis of peritoneal mast cells, and also induced apoptosis of B cells but to a smaller extent, whereas had no effect on T cells, dendritic cells, macrophages, or granulocytes (Additional file 3: Fig. S3). This finding suggests the therapeutic potential of HPE in the treatment of allergic diseases, as the induction of apoptosis in relevant immune cells could improve allergic symptoms by affecting immune responses. However, further research is needed to fully understand the underlying mechanisms.

We observed that the effect of HPE-induced apoptosis on HMC-1 is less significant than in mouse mast cells. Previous studies have indicated that the concentrations of a drug required to induce a similar apoptotic index could vary between human mast lines and murine P815 mastocytoma cell line [38]. This suggests that the response to the same inducer could differ among different cell species.

We evaluated the effect of HPE on mast cell activation using two distinct approaches, i.e., IgE/FcεRI which represents a classical mast cell activation pathway and C48/80, which is an established agent widely used for non-IgE-dependent mast cell activation. We observed HPE-mediated suppression of mast cell activation in both pathways. Therefore, we conclude that HPE maybe a wide-spectrum inhibitor for mast cell activation. Mast cells are hematopoietic cells located at nearly all vascularized tissues and are capable of producing a range of biologically active secreted products, such as various cytokines and growth factors [39]. Our results showed that HPE could inhibit the production of IL-4, IL-6, IL-13 and TNF-α released by mast cells, thus, ultimately, compromised mast cell-mediated allergic diseases. IL-4, IL-6 or TNF-α immunoreactive MCs can be detected using IHC in biopsies of patients with allergic asthma, or atopic dermatitis, [40, 41], and IL-13 exposure could change asthma phenotypes [42], confirming IL-4, IL-6, IL-13 and TNF-α could participate in the pathogenesis of allergic diseases. IL-10 and IL-33 play critical roles in allergic disorders as well. Mast cell-derived IL-10 has been shown to alleviated severe cutaneous contact hypersensitivity reactions [43], and IL-33, an alarming signal in the allergic diseases and inflammation [44], could activate mast cells and trigger downstream reactions. Whether and how HPE affects IL-10 and IL-33 in mast cells await further clarification.

The fact that HPE compromised mast cell migration towards SCF also supports the potential exploitation of HPE in diseases whereby mast cells contribute to pathology. SCF is documented to be not only the major growth factor for mast cells, but is also a chemotactic factor for mast cells. For example, the contribution of mast cells to angiogenesis favouring tumor development has been reported to depend on increased production of SCF by tumor cells [45]. Thus, the possible inhibitory effect of HPE on the regional accumulation of mast cells in the tumor microenvironment may also be explored.

It has been reported that topical application of HPE attenuates the symptoms of DNCB-induced skin lesions, possibly through lowering allergen-specific serum IgE levels and suppressing the production of DNCB-induced reactive oxygen species and oxidative degradation of hyaluronic acid [46]. Furthermore, some natural substances, such as vitamin D, worked similarly as well, which could protect the skin from ultraviolet damage by promoting the production of IL-10 released by mast cells [47]. As mast cells are an important player for the initiation of contact hypersensitivity, we speculate that HPE-mediated suppression of mast cell activities may also be one of the mechanisms accounting for the therapeutic effects of HPE in contact dermatitis.

PCA is an established technique that provides a sensitive in vivo tool to study local mast cell-induced local allergic inflammation. We not only observed compromised dye extravasation after administration of HPE, possibly reflecting antagonized blood vessel permeability increase, but also recorded mast cell degranulation and histamine production at a smaller scale. Thus, the suppressive effects of HPE on PCA may very likely be owing to this molecule’s potential in dampening mast cell activation. Research has found that placental histone was acetylated and dampened the susceptibility to allergic sensitization of kids [48], thus it is possible that the inhibitory effect of HPE in this study may at least partly due to the similar potential. However, the specific regulatory mechanism remains to be fully understood and requires further research.

Given that HPE could induce apoptosis of mast cells, it is possible to question whether the observed effects are due to apoptosis. Although we cannot completely exclude this possibility that the afterwards observations were due to apoptosis induced by HPE, however, we have observed inhibitory effects already after 8 h of HPE treatment, and no obvious cell apoptosis were observed at this time. Therefore, we believe the suppressive effect of HPE on mast cell activation shown in the current study was at least partly not due to apoptosis and further extensively dynamic investigation is needed.

One limitation of this study is that we cannot conclude the precisely active components in the HPE that are responsible for the effect of HPE on mast cells yet. We have previously shown that more than 40 types of functional molecules detectable in the HPE [36], suggesting a comparatively complicated combined- effect might be taken into consideration when further investigations will be proceeded. Meanwhile, we could not prepare a completely adequate control of HPE, such as liver extracts or spleen extracts, thus we used the diluent which dissolves HPE as the control instead. We only focused on the effects of HPE on mast cells in this study, and it will be attractive to expand to other effector cells in allergy, for example, basophils, for their responses to HPE as a future perspective.

## Conclusion

In conclusion, we believe that HPE is capable of exerting a regulatory effect on mast cell biology and may be further exploited for therapeutic or prophylactic treatment strategies for allergic diseases as well as other inflammatory conditions.

### Supplementary Information

**Additional file 1: Fig S1.** The gating strategies of apoptotic mast cells. An example is given to show the gating strategy and the apoptotic cells were revealed by the annexin V^+^ quadrants. (A) C57 cells. (B) HMC-1 cells.

**Additional file 2: Fig S2.** The frequencies of PI^+^ cells and PI^+^ mast cells after HPE treatment. Mice (BALB/c) were challenged intraperitoneally with 120 ng HPE or identical volume of PBS. Peritoneal lavage fluid was collected after 4, 8 or 16 h and the frequencies of PI^+^ cells and PI^+^ mast cells were evaluated with flow cytometric analysis. (A) 4 h. (B) 8 h. (C) 16 h. Data are shown as mean ± SEM (n = 5) of three separate experiments. * P < 0.05, using the unpaired Student t test for statistical significance.

**Additional file 3: Fig S3.** The effect of HPE on the apoptosis of peritoneal immune cells. Mice (BALB/c) were challenged intraperitoneally with 120 ng HPE or identical volume of PBS. Peritoneal lavage fluid was harvested after 16 h and flow cytometry analysis the apoptosis of relevant immune cells. (A) An example is given to show the gating strategy for CD3^+^ T cells and CD19^+^ B cells in each. Cells apoptosis was defined as annexin V^+^ (left panel). The percentages of apoptotic cells were plotted in (B) T cells (CD3^+^ cells), (C) B cells (CD19^+^ cells), (D) Dendritic cells (CD11c^+^ cells), (E) Macrophages (F4/80^+^ cells) and (F) Granulocytes (Gr-1^+^ cells). Data are shown as mean ± SEM (n = 5) of three separate experiments. * *P < 0.01, using the unpaired Student t test for statistical significance.

## Data Availability

The datasets used or analyzed during the current study are available from the corresponding author on reasonable request.

## Abbreviations

HPE: Human placental extract
PCA: Passive cutaneous anaphylaxis
GVHD: Graft-versus-host diseases
CCK8: Cell Counting Kit-8
β-hex: β-Hexosaminidase
PLF: Peritoneal lavage fluid
C48/80: Compound 48/80
