# Supplementary figures and images for: Human placental extract suppresses mast cell activation and induces mast cell apoptosis
Source: Allergy Asthma Clin Immunol. 2023 Nov 27;19:98. doi: 10.1186/s13223-023-00850-y (PMC10683163; doi:10.1186/s13223-023-00850-y)

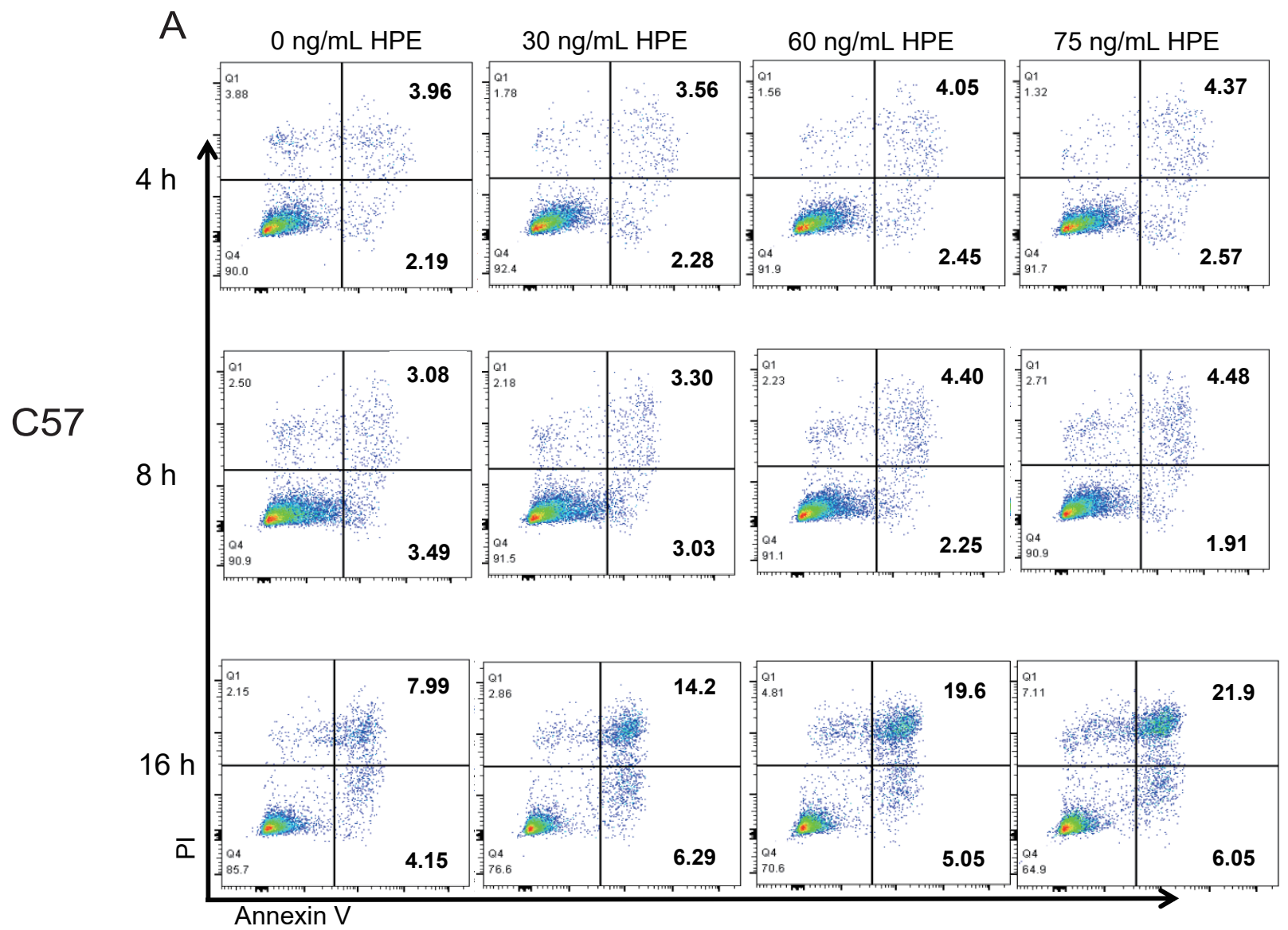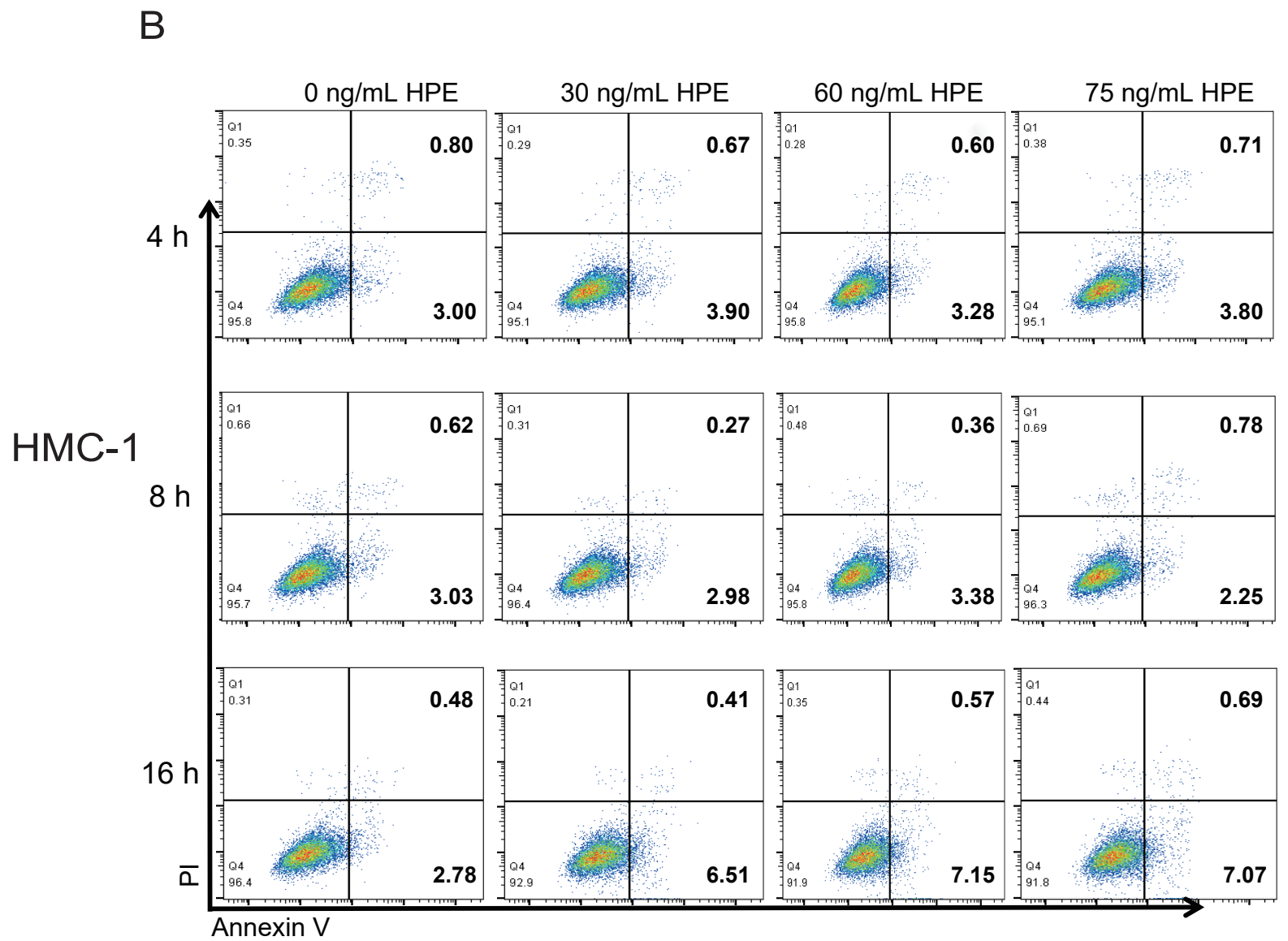

Supplement: Supplementary file 1 — Additional file 1: Fig S1. The gating strategies of apoptotic mast cells. An example is given to show the gating strategy and the apoptotic cells were revealed by the annexin V+ quadrants. (A) C57 cells. (B) HMC-1 cells. [file 13223_2023_850_MOESM1_ESM.pdf]

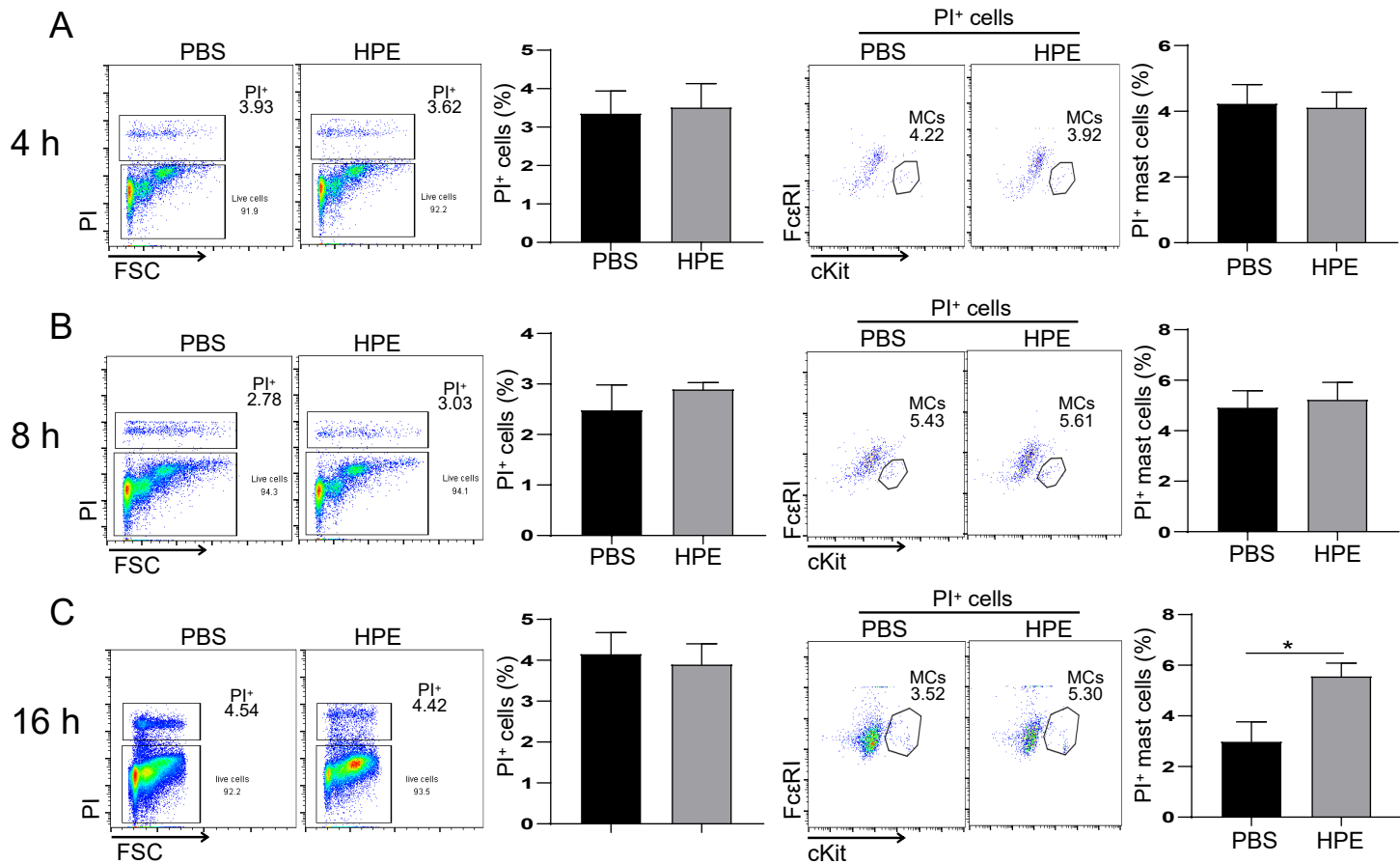

Supplement: Supplementary file 2 — Additional file 2: Fig S2. The frequencies of PI+ cells and PI+ mast cells after HPE treatment. Mice (BALB/c) were challenged intraperitoneally with 120 ng HPE or identical volume of PBS. Peritoneal lavage fluid was collected after 4, 8 or 16 h and the frequencies of PI+ cells and PI+ mast cells were evaluated with flow cytometric analysis. (A) 4 h. (B) 8 h. (C) 16 h. Data are shown as mean ± SEM (n = 5) of three separate experiments. * P < 0.05, using the unpaired Student t test for statistical significance. [file 13223_2023_850_MOESM2_ESM.pdf]

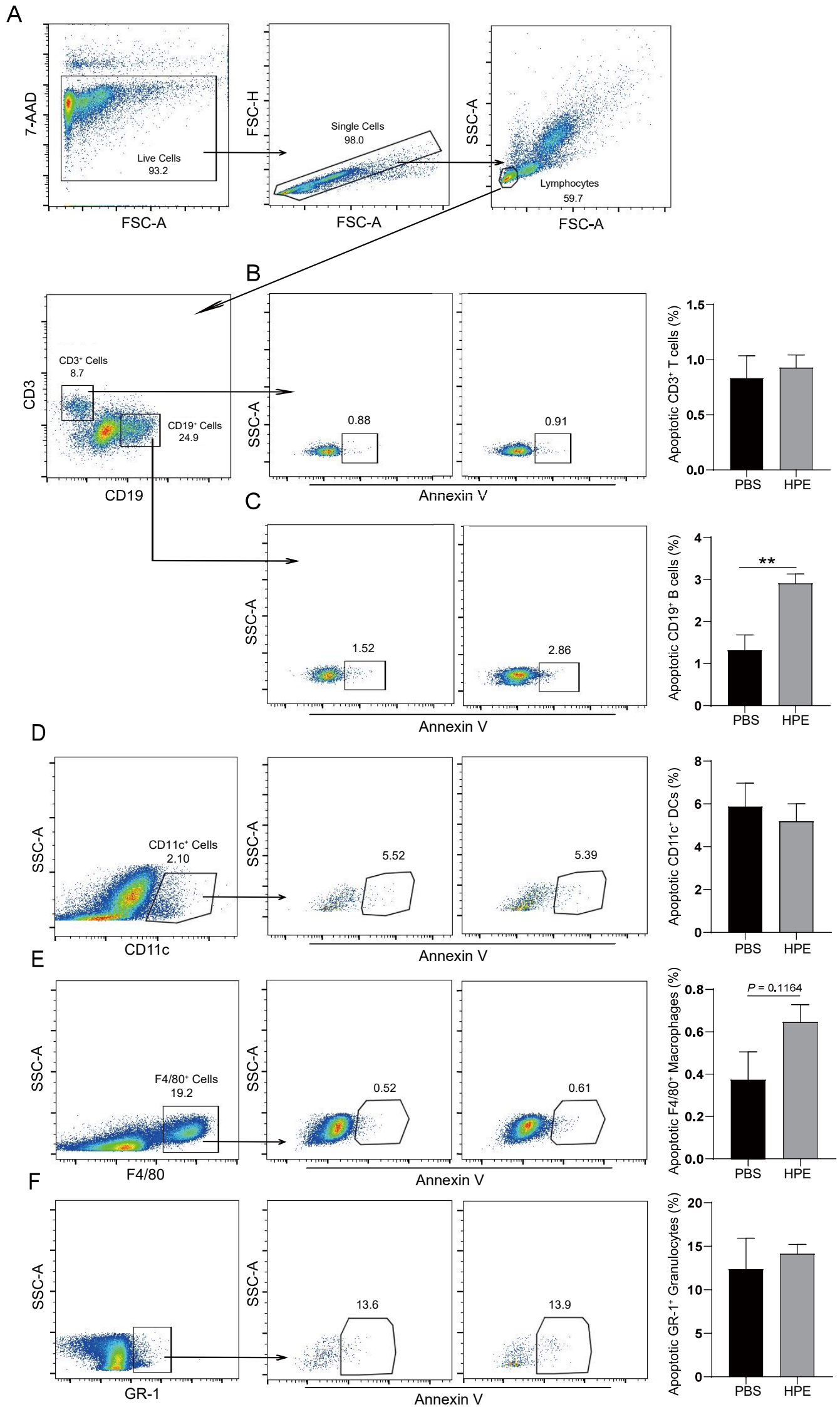

Supplement: Supplementary file 3 — Additional file 3: Fig S3. The effect of HPE on the apoptosis of peritoneal immune cells. Mice (BALB/c) were challenged intraperitoneally with 120 ng HPE or identical volume of PBS. Peritoneal lavage fluid was harvested after 16 h and flow cytometry analysis the apoptosis of relevant immune cells. (A) An example is given to show the gating strategy for CD3+ T cells and CD19+ B cells in each. Cells apoptosis was defined as annexin V+ (left panel). The percentages of apoptotic cells were plotted in (B) T cells (CD3+ cells), (C) B cells (CD19+ cells), (D) Dendritic cells (CD11c+ cells), (E) Macrophages (F4/80+ cells) and (F) Granulocytes (Gr-1+ cells). Data are shown as mean ± SEM (n = 5) of three separate experiments. * *P < 0.01, using the unpaired Student t test for statistical significance. [file 13223_2023_850_MOESM3_ESM.pdf]
